# Designing Spoonable Milk Kefir Gels: From Fermentation Optimization to Clean-Label Gel Structuring with Psyllium

**DOI:** 10.3390/gels11090693

**Published:** 2025-09-01

**Authors:** María Cardenete-Fernández, Alicia Castillo-Rivas, M. Montaña Durán-Barrantes, Luis A. Trujillo-Cayado, Jenifer Santos

**Affiliations:** 1 Departamento de Ingeniería Química, Facultad de Química, Universidad de Sevilla, c/Profesor García González, 41012 Sevilla, Spain; mariacardenetefernandez@gmail.com (M.C.-F.); acastillo6@us.es (A.C.-R.); mmduran@us.es (M.M.D.-B.); 2 Departamento de Ingeniería Química, Escuela Politécnica Superior, Universidad de Sevilla, c/Virgen, de África 7, 41011 Sevilla, Spain; 3 Facultad de Ciencias de la Salud, Universidad Loyola Andalucía, Avda. de las Universidades s/n, Dos Hermanas, 41704 Sevilla, Spain; jsgarcia@uloyola.es

**Keywords:** fermented dairy product, functional food, hydrocolloid, probiotic beverage, rheology

## Abstract

Kefir is a fermented dairy product whose structural properties can be modified to enhance its nutritional and sensory profile. The objective of this study was to develop spoonable milk kefir gels by optimizing fermentation conditions and incorporating psyllium and calcium chloride as structuring agents. In the initial phase of the study, a full factorial design was employed to conduct a comparative analysis of whole milk and skimmed milk during the fermentation process of kefir. The study encompassed the evaluation of the impact of various parameters, including inoculum level, temperature, and fermentation time, on the acidification kinetics of the fermentation process. This evaluation was facilitated through the measurement of pH and total acidity levels. Skimmed milk demonstrated accelerated acidification, consistently attaining a final pH of 4.08 and a total acidity of 9.99 g·L^−1^ lactic acid equivalents under optimized conditions (5.5% weight:weight grains, 26 °C, 24 h). In the subsequent phase, kefir obtained under these conditions was gelled with varying concentrations of psyllium and calcium chloride. Rheological characterization revealed that psyllium markedly strengthened the gel network: at 3.06% *w*:*w* psyllium, the elastic modulus increased up to 209.6 Pa, while the critical stress improved from 0.64 Pa at low psyllium/Ca^2+^ to 10.42 Pa at high psyllium content. Furthermore, zero-shear viscosity increased substantially, exceeding 1500 Pa·s in high-psyllium, low-calcium formulations. The findings demonstrate that combining fermentation optimization with clean-label structuring agents enables the development of low-fat kefir gels with enhanced textural and processing properties, supporting their potential as synbiotic, functional dairy products.

## 1. Introduction

Milk kefir is a traditional fermented milk beverage produced by inoculating milk with kefir “grains”, a symbiotic consortium of lactic acid bacteria, yeasts, and acetic acid bacteria embedded in a polysaccharide–protein matrix known as kefiran. The fermentation process yields a mildly acidic, slightly effervescent drink with a characteristic flowing consistency (pourable viscosity) rather than a firm gel [1]. Kefir has attracted interest for its probiotic qualities and health benefits; studies have reported improved lactose digestion, modulation of cholesterol metabolism, immune support, and other positive effects from regular kefir consumption [2]. From a nutritional perspective, milk kefir is characterized by its high protein content. The vitamin content of kefir is also significant, with folic acid and B vitamins being particularly noteworthy [3]. In addition to these nutrients, kefir is rich in minerals such as calcium and phosphorus, which are essential for various bodily functions [4]. Kefir is also a source of micronutrients such as calcium and vitamins. However, the levels of these micronutrients present in the beverage are highly dependent on the composition of the milk substrate employed in its production. Indeed, whole milk and skimmed milk differ in both concentration and bioavailability. Beyond its nutritional profile, the present study emphasizes the development of kefir gels with a “spoonable” texture. In terms of rheology, spoonable gels are defined by a solid-like viscoelastic behavior, where the storage modulus (G′) exceeds the loss modulus (G″), in combination with the presence of a measurable yield stress and sufficiently high apparent viscosity to maintain shape, yet low enough to allow easy consumption with a spoon. The product is characterized in these terms, thus highlighting its functional nature and providing a direct link between the technological objectives and the rheological parameters assessed in this work. In order to expand the application of kefir as a functional foodstuff, researchers are exploring methods of transforming kefir into spoonable or gelled products without compromising its probiotic integrity [5,6].

The production of kefir is traditionally accomplished through the fermentation of milk by kefir grains, which consist of a complex symbiotic matrix of lactic acid bacteria (LAB), acetic acid bacteria, and yeasts embedded in a polysaccharide-protein matrix primarily composed of kefiran [7]. This microbial consortium initiates multiple simultaneous metabolic pathways, including glycolysis, proteolysis, and lipolysis, resulting in a wide range of metabolites such as lactic acid, ethanol, carbon dioxide, and various aroma compounds. It is imperative to note that, amongst these factors, the progressive accumulation of organic acids (primarily lactic and acetic acids) assumes particular significance for both the sensory characteristics and the microbiological safety of the final product. Monitoring the pH level is of paramount importance during the fermentation process of kefir, as it undergoes a decline in pH as the process of acid production intensifies [8]. The decline in pH has been demonstrated to play a pivotal role in the process of protein aggregation and the destabilization of casein micelles. These phenomena are of paramount importance in the context of the development of viscosity and the potential gelation that occurs within fermented milk systems. Concurrently, total acidity (TA) is used to estimate the concentration of free and bound protons, thereby functioning as a complementary indicator of the extent of fermentation. Whilst pH is indicative of the present hydrogen ion concentration, TA provides a more comprehensive measure of acid content and buffering capacity. These are both essential for controlling the fermentation process and maintaining microbial viability. The utilization of skimmed milk as a fermentation substrate carries with it specific ramifications for the production of kefir. Skimmed milk, being low in fat, has been shown to possess a reduced buffering capacity in comparison with whole milk. This reduced capacity can result in an accelerated rate of pH decrease during the fermentation process. Furthermore, the absence of milk fat alters the availability of certain substrates and may modulate the metabolic activity of the microbial consortium. Nonetheless, earlier research has demonstrated that LAB and yeasts exhibit a high degree of adaptability in skimmed milk environments [9], achieving adequate acidification and microbial growth to yield a stable and functional kefir product. Despite the fact that the absence of fat may result in kefir with reduced viscosity and diminished creamy mouthfeel, these sensory limitations can be addressed by incorporating dietary fibers or hydrocolloids, which enhance texture and water retention without compromising fermentation [10]. It is important to note that the successful development of kefir gels using skimmed milk offers a promising avenue for creating low-fat, functional fermented products with enhanced nutritional value. In this context, it is imperative to comprehend the acidification kinetics and pH behavior during the fermentation process, as these parameters exert a direct influence on the structural development and the eventual rheological properties of the product.

One way to create a gel-like structure in kefir is to add natural hydrocolloid fibers, which thicken or gel the aqueous phase. Building on this concept, we propose using psyllium husk as a gelling agent in kefir. Psyllium (derived from *Plantago ovata* seeds) is a soluble dietary fiber renowned for its exceptional water-holding and gel-forming capacity. Psyllium husk is chemically rich in highly branched arabinoxylans (largely composed of xylose and arabinose sugars), which form viscous mucilage when hydrated [11]. Psyllium can create a strong gel network due to its long-chain, cross-linkable polymer structure [12]. This gel-forming property of psyllium is closely tied to its health benefits, which have earned psyllium recognition as a functional fiber, approved by regulatory agencies for health claims [13]. Given that the health benefits of psyllium are well established, as are those of kefir, we will focus on its role as a clean-label, gel-forming fiber that gives kefir its spoonable, functional, probiotic texture. The study therefore centers on fermentation optimization and rheological characterization rather than bioactivity testing. This study also examines the addition of calcium chloride (CaCl_2_) to further enhance the gel structure of a kefir–psyllium system. Calcium ions (Ca^2+^) are well known to strengthen gel networks in various food systems by acting as cross-linking agents. Calcium fortification has also been reported to increase the firmness and viscosity of fermented milk gels such as yoghurt, although the effect can depend on the form and timing of calcium addition [14]. In the context of polysaccharide gels, Ca^2+^ can interact with negatively charged functional groups on polymer chains, creating cross-links between chains. Psyllium’s chemical structure includes uronic acids or other sites that can bind cations, and the presence of Ca^2+^ dramatically influences psyllium gel properties [15].

Psyllium’s gel-forming ability has been extensively linked to its high content of soluble arabinoxylans, which form cohesive, water-rich networks through extensive hydration, chain entanglement, and intermolecular hydrogen bonding [16,17]. While psyllium alone forms relatively weak gels, its combination with other biopolymers under controlled pH and ionic conditions can yield denser and more stable hydrogel structures [17]. In the presence of Ca^2+^, negatively charged uronic acid residues and ferulic-acid-substituted side chains on arabinoxylans can participate in ionic cross-linking, thereby increasing elastic modulus, altering microstructure, and improving thermal resilience [15,18]. However, excessive Ca^2+^ may lead to charge screening and coil compression, causing partial network collapse and reduced gel stiffness. From a nutritional perspective, human trials indicate that typical psyllium doses have only a marginal effect on calcium absorption [19], although very high intakes can reduce bioavailability in animal models [20]. This dual technological–nutritional perspective provides the rationale for investigating psyllium–Ca^2+^ interactions in kefir-based gels.

A fundamental element of this research endeavor pertains to the rheological characterization of the kefir formulations, with the objective of quantifying their gel properties and flow behavior. Rheology is a critical tool in the field of food science, as it enables the correlation of composition and microstructure to texture and stability [21]. Researchers have noted that rheological measurements are essential for the development and optimization of food products, given their impact on processing and consumer perception [22]. In this study, we utilize small-amplitude oscillatory shear tests and steady shear flow tests to evaluate the viscoelastic and flow characteristics of kefir with added psyllium and CaCl_2_. Oscillatory stress sweeps (or strain sweeps) are performed to identify the linear viscoelastic region (LVR) and determine the yield stress/strain at which the gel structure begins to break down. Frequency sweep tests (within the LVR) allow for the measurement of the storage modulus G′ (elastic response) and loss modulus G″ (viscous response) across a range of frequencies. This reveals the material’s gel-like nature and structural stability. In order to form a gel, it is expected that G′ will dominate over G″ (G′ >> G″), and that there will be little frequency dependence, indicating solid-like behavior.

Therefore, the present study aimed to (i) investigate the fermentation kinetics of skimmed milk kefir under different process conditions, (ii) include whole milk as a reference matrix to quantify the influence of fat content on acidification behavior and to validate the transferability of optimal fermentation parameters to the low-fat substrate, and (iii) apply the selected conditions to produce spoonable kefir gels by incorporating psyllium husk and calcium chloride. This sequential approach ensures that the optimized low-fat formulation is grounded on a robust comparison with a widely used full-fat benchmark, thereby enhancing the relevance and reproducibility of the findings.

## 2. Results and Discussion

### 2.1. Optimization of Fermentation to Define the Gelation Baseline

A comparative study was conducted to evaluate the effects of fermentation conditions, namely time (t), temperature (T), and kefir grain concentration (g), on the physicochemical properties of kefir made from whole and skimmed milk. The two response variables were: pH and total acidity (expressed as lactic acid equivalents).

As anticipated, a decline in pH was evident in conjunction with escalating fermentation intensity, characterized by elevated grain percentages, augmented temperatures, and protracted fermentation durations (see Table 1). The lowest pH values were found in experiment 8 (48 h, 26 °C, 10% (*w*:*w*)), indicating intense fermentation, while the highest pH values were observed in experiment 1 (12 h, 20 °C, 1% (*w*:*w*)), under milder fermentation conditions. The pH trajectories exhibited by both skimmed and whole milk kefir samples were comparable, indicating that the fat content does not exert a substantial influence on the acidification kinetics. It is noteworthy that kefir samples attained safe consumption pH values (≤4.5) within the initial 24 h under sufficiently intense fermentation conditions. It is evident from the data that there was a clear upward trend in total acidity, which was concomitant with an increase in fermentation intensity. Experiment 8 yielded the highest acidity values, while Experiment 1 yielded the lowest. Mid-range values were recorded in the two central-point replicates (experiments 9 and 10). A comparative analysis of the acidity profiles revealed that both milk types exhibited comparable outcomes, though a greater degree of variability was observed in whole milk. This variability may be attributed to the complex buffering and particulate nature of the medium. Notably, an inverse and non-linear relationship was observed between TA and pH, consistent with the logarithmic nature of pH but moderated by the buffering capacity of milk proteins and salts. This finding underscores the notion that acidity perception is more closely associated with total acid content than with pH alone. This finding serves to reinforce the feasibility of utilizing skimmed milk in the production of kefir, even in cases where grains have been originally adapted to whole milk. The fermentation kinetics are influenced by milk composition, where protein, fat, and carbohydrate fractions jointly contribute to the buffering environment and microbial activity.

A full factorial statistical analysis was conducted to quantitatively assess the influence of fermentation parameters on the physicochemical properties of kefir. The models were developed independently for kefir produced with skimmed and whole milk. For skimmed milk kefir, the regression equation for pH indicated that all three variables contributed to its reduction, with fermentation time being the most influential factor, as evidenced by its largest negative coefficient (−0.61). The model demonstrated a robust fit, with an R^2^ value of 0.903 and an adjusted R^2^ of 0.854, indicating that the variability in pH was effectively explained by the experimental factors. The response surface plots confirmed this trend: an increase in either fermentation time or temperature resulted in a progressive decrease in pH, reflecting the intensified acidification process. In a similar manner, an increase in kefir grain percentage contributed to further acidification, albeit to a lesser extent.

(1) pH=4.65−0.32×g−0.39×T−0.61×t

When evaluating whole milk kefir, a similar model was obtained, with fermentation time again emerging as the most significant variable (coefficient = −0.74). The statistical robustness of the model was confirmed by R^2^ and adjusted R^2^ values of 0.921 and 0.882, respectively. Response surface analyses revealed a consistent reduction in pH with increasing values of all three independent variables. These findings underscore the observation that, irrespective of the fat content of the milk, fermentation kinetics in terms of acidification exhibit analogous trends. The comparable behavior exhibited by both milk types indicates that milk fat does not significantly influence microbial acid production or metabolic activity during the fermentation process. This observation is consistent with previous reports that highlight the role of overall milk composition in modulating acidification rates during LAB fermentation.

(2) pH=4.68−0.28×g−0.36×T−0.74×t

With regard to total acidity (TA), the regression model for skimmed milk kefir demonstrated that all three variables (fermentation time, temperature, and grain percentage) exerted a positive effect on acidity, with fermentation time once again proving to be the most dominant. The model demonstrated an optimal fit (R^2^ = 0.990; adjusted R^2^ = 0.969), signifying elevated predictive reliability. The response surfaces demonstrated that an increase in fermentation time resulted in a significant rise in acid production, with temperature and grain concentration exerting secondary, but nevertheless notable, effects.

(3) TA(gL)=7.50+2.28×g+2.13×T+4.16×t

In the case of kefir produced using whole milk, the acidity model exhibited a comparable structure, with time demonstrating the most significant positive coefficient. In contrast to the findings of the pH models, interaction terms between variables were found to be statistically significant, suggesting a more complex interplay in the development of acidity. The model demonstrated robust performance, evidenced by R^2^ = 0.992 and adjusted R^2^ = 0.975. The response surfaces revealed that total acidity increased in proportion to the rise in fermentation time, temperature, and grain concentration. These patterns were consistent with the experimental data and mirrored those observed for skimmed milk kefir, thereby reinforcing the hypothesis that the milk’s fat content does not notably affect microbial acidification dynamics.

(4) TA(gL)=7.77+2.38×g+2.90×T+4.47×t+1.03×g×T+1.27×g×t+1.80×T×t

This trend further confirms the non-linear inverse dependence of pH on lactic acid equivalents, reflecting the buffering effects of the milk matrix. While pH is indicative of the concentration of free protons in the medium, total acidity quantifies both free and bound protons from organic acids. In this context, an observation was made of a gradual decline in pH, attributable to the buffering capacity of milk, which is primarily ascribed to proteins and mineral salts. These substances neutralize a proportion of the acid load in the initial phase. Conversely, total acidity exhibited a more marked and linear increase over time, providing a more sensitive indicator of microbial metabolic activity.

The statistical analysis confirmed that fermentation time is the most critical factor influencing both pH and total acidity of kefir, followed by temperature and kefir grain concentration. Such behavior reinforces that milk constituents (including fat) affect fermentation indirectly by altering the physicochemical environment, rather than fat acting as a direct substrate. This finding supports the use of skimmed milk in experimental designs targeting low-fat kefir formulations.

The fermentation condition that was optimized in this study (5.5% kefir grains, 26 °C, 24 h) consistently yielded a final pH of approximately 4.08 and a total acidity of about 10 g L^−1^ lactic acid equivalents. The selection of this standardized endpoint was driven by the necessity to ensure reproducibility and to establish a consistent physicochemical baseline for all subsequent gelation experiments. The effect of psyllium and CaCl_2_ on the gel structure was assessed in the absence of interference from uncontrolled acidification by controlling the fermentation state. Variations in casein charge, calcium activity and serum composition were also controlled.

### 2.2. Gel Formation and Rheological Characterization

All gelation assays were performed using kefir produced under the optimized fermentation conditions described in Section 2.1, ensuring identical initial pH, total acidity, and microstructural state across samples. Standardizing these parameters has been shown to control key variables such as casein micelle charge, ionic strength, and calcium partitioning. These, in turn, have been demonstrated to directly influence the interactions between psyllium, calcium ions, and the protein–fat matrix.

The second experimental design sought to evaluate the impact of psyllium and calcium chloride (CaCl_2_) concentrations on the physicochemical characteristics of skimmed milk kefir. The experimental phase was developed using the fermentation conditions that had been optimized in the preliminary design, which were as follows: 5.5% kefir grains, 26 °C, and 24 h of fermentation. The selection of these conditions was driven by the objective of achieving a balance between sufficient microbial activity and sensory acceptability, thereby avoiding the elevated acidity levels that have been previously associated with extended fermentation times. In order to establish a baseline, the “blank sample” (kefir produced under the aforementioned conditions but without the addition of psyllium or calcium) was evaluated. The blank sample exhibited a pH of 4.08 ± 0.02 and a total acidity of 9.99 ± 0.09 g lactic acid·L^−1^, which is in accordance with the expected values for well-fermented kefir. This finding serves to confirm the suitability and reproducibility of the chosen fermentation conditions. Subsequently, the effect of psyllium and CaCl_2_ incorporation on the pH of kefir was investigated. pH measurements were conducted both immediately after the addition of the additives (t = 0) and after a two-hour hydration period. Across the three samples analyzed, only minor variations were observed. In two cases, a slight increase in pH was detected (e.g., from 4.10 to 4.20 and from 4.11 to 4.23), whereas the third sample remained essentially stable. The findings indicate that neither psyllium nor CaCl_2_ caused a substantial acidification or neutralization of the matrix within the two-hour period. This stability is of technological interest, as it implies that the incorporation of these additives does not adversely alter the acidity profile of the final product, thereby maintaining its characteristic sensory attributes.

**Figure 1:**
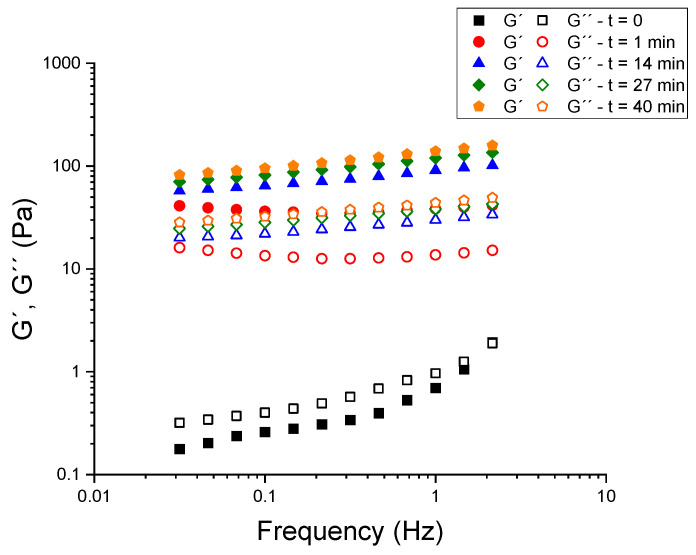
Mechanical spectra for a kefir sample with 0.0375% (*w*:*w*) CaCl_2_ and 2% (*w*:*w*) psyllium as a function of gelation time.

The rheology of skimmed-milk kefir systems formulated with psyllium and calcium chloride (CaCl_2_) was investigated to quantify the modulation of small-amplitude structure and flow behavior by these factors. Prior to the comparison of formulations within the experimental design, a robust hydration protocol for psyllium was established, given that its water uptake kinetics strongly influence the reproducibility of viscoelastic measurements. Mechanical spectra, defined as frequency sweeps at constant stress, are shown in Figure 1 for a kefir blank sample (26 °C, 24 h, 5.5% grains) containing 0.0375% (*w*:*w*) CaCl_2_ and 2% (*w*:*w*) psyllium. The experiment was conducted in two phases: initially, the kefir base was subjected to stress without the addition of psyllium (t = 0); subsequently, the kefir base was subjected to stress after the addition of 2% (*w*:*w*) psyllium. Tests were conducted at a constant shear stress of 0.2 Pa to ensure operation within the linear viscoelastic region (LVR). In the absence of psyllium, the kefir exhibited liquid-like behavior (G″ > G′ across most of the spectrum). It is noteworthy that, in a striking demonstration of rapidity, the ordering underwent an inversion a mere minute following the addition of psyllium (G′ > G″ at all frequencies that were examined), accompanied by a surge in both moduli that exceeded an order of magnitude. The progressive growth of G′ and G″ continued for several tens of minutes; beyond approximately 40 min, the spectra no longer evolved beyond the experimental error. Consequently, a 45-min rest period was implemented for all subsequent measurements to ensure complete and reproducible hydration of psyllium prior to rheological testing.

**Figure 2:**
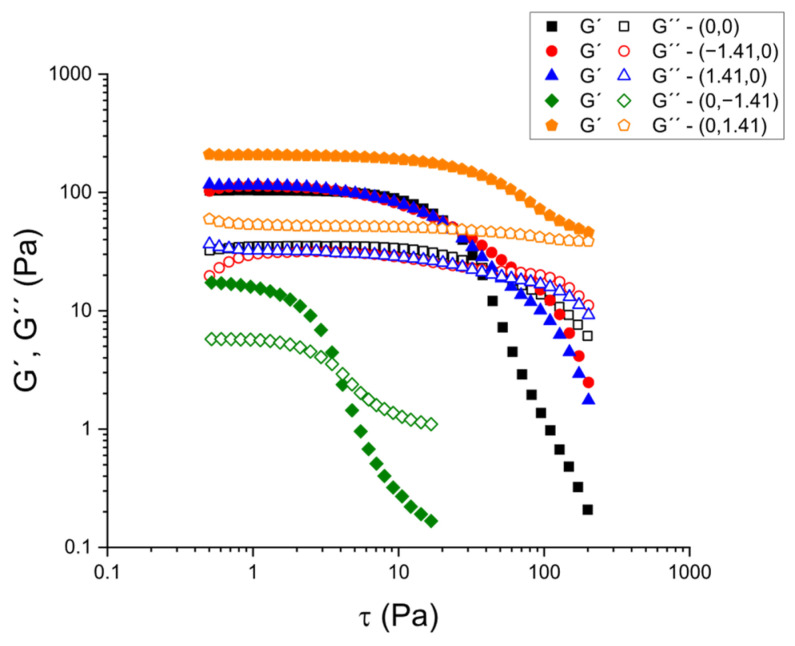
Stress sweeps for selected kefir samples as a function of psyllium and CaCl_2_ concentration.

Subsequently, stress sweep tests were performed at 1 Hz to identify the LVR and determine the critical stress (τ_c_) at which the microstructure begins to break down. Figure 2 shows stress scans for selected samples based on calcium chloride and psyllium concentrations, in accordance with the experimental design. In the range of CaCl_2_ and psyllium concentrations that were examined, all samples exhibited a measurable LVR, wherein G′ and G″ remained virtually constant with increasing stress. This was followed by a sharp decrease in G′, indicating the onset of irreversible structural breakdown. The amplitude of the LVR and the magnitude of τ_c_ provide complementary metrics of the robustness of the network under processing-like disturbances (filling, pumping, vibration), while G′ in the LVR (G′_LVR_) measures the small-deformation stiffness of the gelatinous matrix.

A response surface analysis (RSM) was performed using psyllium and CaCl_2_ concentrations as independent variables and τc, G′_LVR_, and the loss tangent at 1 Hz as responses (see Table 2).

The loss tangent (tan δ) is defined as the ratio of the loss to the storage modulus (=G″/G′). It has been demonstrated that this parameter quantifies the balance between viscous dissipation and elastic energy storage. Values below 1 indicate an elastic dominance, and, consequently, predominantly solid-like (gel) behavior. The analysis of the results yielded three empirical equations, based on a quadratic model, which relate these rheological parameters to the concentrations of calcium chloride (CaCl_2_) and psyllium (Psy):

(5) $$\tau_{c}(Pa)=3.54+2.98\times Psy+0.91\times {Psy}^{2}$$

(6) $${G^{\prime }}_{RVL}(Pa)=128.9+72.2\cdot Psy-57.8\cdot {CaCl}_{2}\cdot Psy$$

(7) $$\tan\delta =0.26-0.01\cdot {CaCl}_{2}-0.03\cdot Psy+0.02\cdot {CaCl}_{2}\cdot Psy+0.01\cdot {\left({CaCl}_{2}\right)}^{2}+0.02\cdot {\left(Psy\right)}^{2}$$

**Figure 3:**
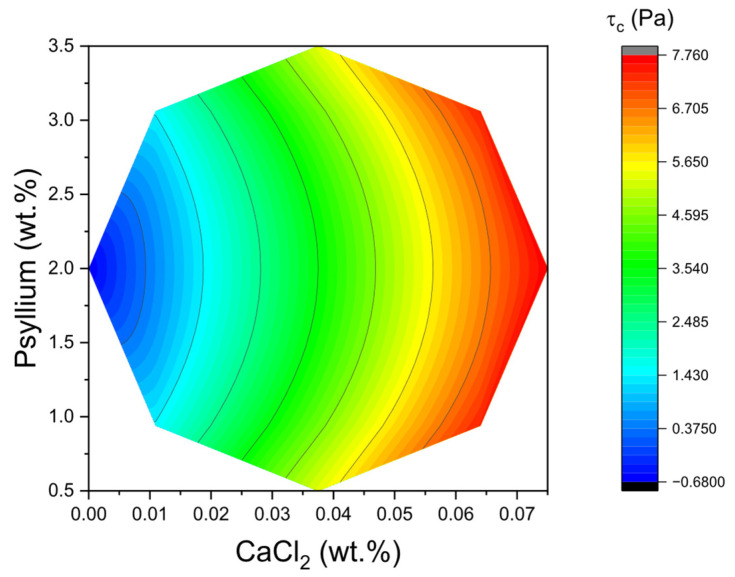
Two-dimensional response surface of critical stress (τ_c_) values as a function of CaCl_2_ concentration and psyllium.

These models also demonstrated robust performance, evidenced by R^2^ = 0.971 and adjusted R^2^ = 0.939, R^2^ = 0.959 and adjusted R^2^ = 0.908, and R^2^ = 0.919 and adjusted R^2^ = 0.818, respectively. For a constant concentration of CaCl_2_, an increase in psyllium resulted in elevated values of both G′_LVR_ and τ_c_. In stress sweeps, this manifested as higher plateau G′ in the LVR and a rightward shift of the yielding point, indicating a denser and more cohesive network capable of withstanding larger imposed stresses before catastrophic softening. The two-dimensional surface for τ_c_ shown in Figure 3 corroborates the primacy of psyllium concentration as the main lever to extend the LVR and delay yielding. In practical terms, formulating towards the higher end of the psyllium range systematically enlarged τ_c_, even when CaCl_2_ was held constant. The physical interpretation is consistent with the high molecular weight and pronounced water-binding capacity of psyllium arabinoxylans, which promote chain entanglement and interparticle bridging in the protein–polysaccharide–fat droplet continuum of kefir.

**Figure 4:**
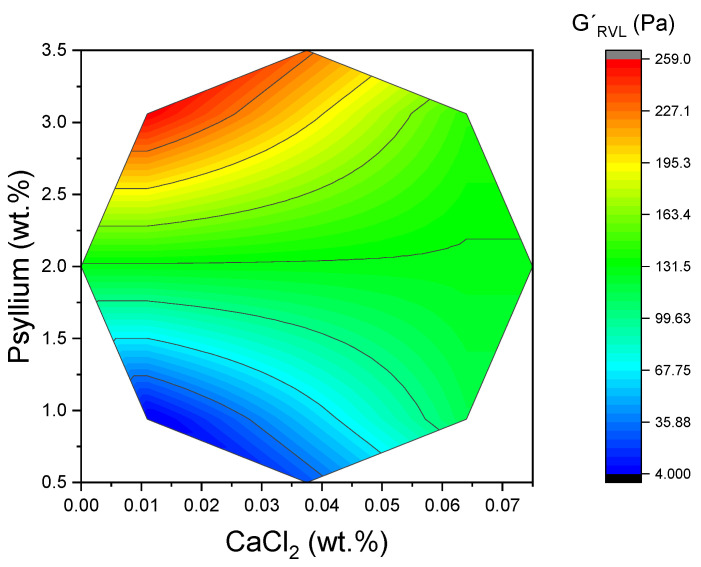
Two-dimensional response surface of elastic modulus at linear viscoelastic range (G′_LVR_) values as a function of CaCl_2_ concentration and psyllium.

Furthermore, Figure 4 demonstrates the two-dimensional surface area for G′_LVR_, illustrating the impact of both variables, particularly psyllium. The resulting increase in junction density and network connectivity has been shown to boost elastic energy storage under small deformations and to delay the strain-amplified rupture processes that define τ_c_. From a product perspective, higher G′_LVR_ implies firmer spoonable texture, and higher τ_c_ suggests improved resilience during handling, transport, and oral processing. The influence of CaCl_2_ was contingent upon the psyllium level. The response surfaces indicated that the maximum G′_LVR_ occurred at elevated psyllium contents and comparatively low CaCl_2_ levels. Further increases in CaCl_2_ tended to reduce G′_LVR_, despite the presence of substantial polysaccharide. Consequently, the G′ parameter within the LVR experiences a decline, indicating a reduction in the system’s ability to resist deformation. As demonstrated in Figure 1, there was also a decline in the τ_c_ from 10.42 Pa to 3.39 Pa at 3.06% (*w*:*w*) psyllium. Conversely, at low psyllium content (0.94% (*w*:*w*)), τ_c_ increased from 0.64 Pa to 3.68 Pa as the concentration of Ca^2+^ increased, suggesting limited ionic bridging under dilute conditions. The effects observed in this study, which were found to be both coordinated and factor-dependent, align with prior reports of divalent-ion-induced polysaccharide coil contraction and network heterogeneity [23,24]. Whilst direct microstructural visualization was not a primary objective of the present study, the internal consistency of the rheological data provides a robust foundation for the interpretation proposed herein.

**Figure 5:**
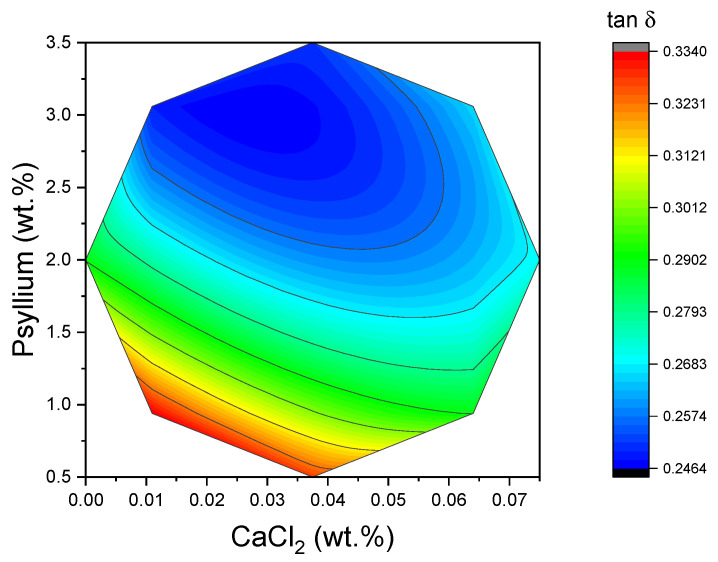
Two-dimensional response surface of tan δ values as a function of CaCl_2_ concentration and psyllium.

The loss tangent integrates the elastic and viscous contributions into a single, dimensionless descriptor of “gel character.” In the context of food science, low tan δ values (less than 0.1) are indicative of strong, highly elastic gels, while values approaching unity suggest weak gels or paste-like materials. In the present study, tan δ values were found to be comparatively low and concentrated within a narrow window (0.24–0.33), with the lowest values being observed at high psyllium/low CaCl_2_ levels (see Figure 5). This region coincided with the peak of G′_LVR_. This alignment of low tan δ with high G′_LVR_ is consistent with the formation of a well-connected, elastic network. While the tan δ surface was mathematically more complex than those of G′_LVR_ and τc, its limited spread underscores that, within the design space explored, all formulations behaved as gels rather than viscous suspensions. These interpretations are in accordance with standard rheological practice, whereby tan δ is utilized as a rapid indicator of gel strength and water-holding ability in structured foods.

**Figure 6:**
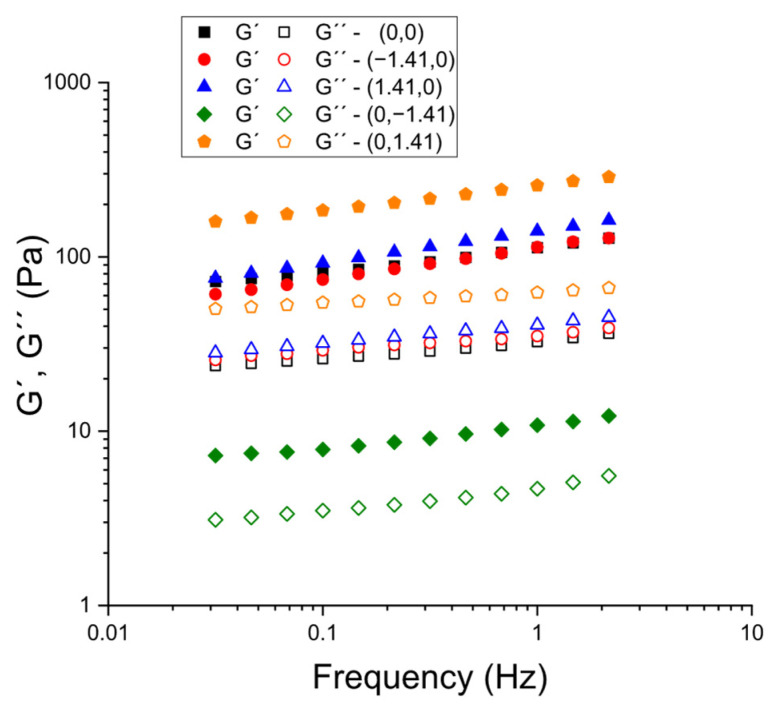
Frequency sweeps for selected kefir samples as a function of psyllium and CaCl_2_ concentration.

As illustrated in Figure 6, a series of frequency sweeps were conducted on specific formulations (selected to ascertain the impact of psyllium at constant CaCl_2_ and vice versa). Methodologically, the stress amplitude in the frequency sweeps was kept constant within the LVR identified for each sample, following the protocol validated in the hydration-time study (constant-stress frequency sweeps after ensuring the LVR by a prior stress sweep). This ensured that the frequency dependence of G′ and G″ reflected material structure rather than progressive damage during testing. Across all compositions that were analyzed, the storage modulus exceeded the loss modulus throughout the explored frequency window (G′ > G″), and G′ demonstrated only a weak frequency dependence, which is characteristic of weak-gel behavior. In accordance with the earlier determination of tan δ at 1 Hz surface, the spectra position the formulations towards the ‘elastic-dominant’ side of viscoelasticity, with reduced tan δ (and consequently augmented gel character) systematically observed at elevated levels of psyllium and reduced levels of CaCl_2_. These mechanical spectra thus corroborate the hypothesis that the formulations form percolated networks at small deformations, and that increasing psyllium primarily strengthens the network. Conversely, Ca^2+^ additions in the studied range tend to attenuate the elastic response.

**Figure 7:**
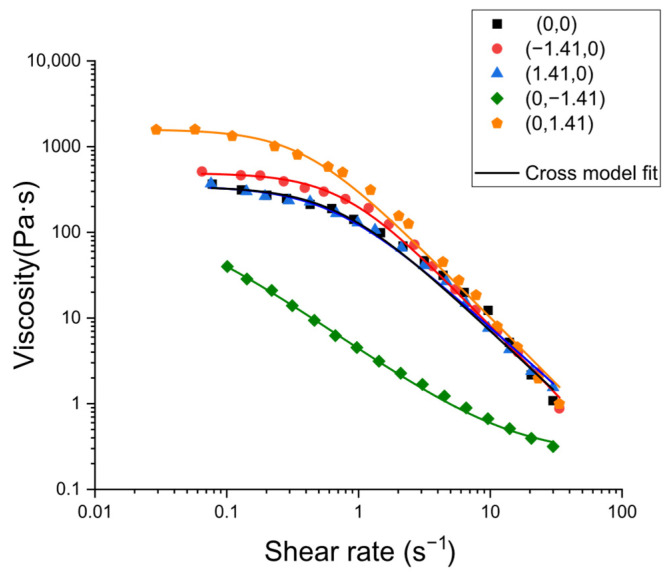
Flow curves for selected kefir samples as a function of psyllium and CaCl_2_ concentration.

The analysis of flow curves illustrated in Figure 7 (apparent viscosity versus shear rate) revealed pronounced shear-thinning behavior in all samples. This behavior is dominated by progressive chain disentanglement and structural alignment under flow. The Cross model provided a comprehensive description of all datasets, whereby the transition of viscosity between a zero-shear plateau (η_0_) and a high-shear plateau (η_∞_) occurred, accompanied by a characteristic time constant t (1/t marking the initiation of the power-law regime) and a shear-thinning index m determining the gradient of the intermediate region:

(8) $$\eta =\eta_{\infty }+\frac{\eta_{0}-\eta_{\infty }}{{1+(t\times \dot{\gamma })}^{m}}$$

The favorable agreement with Cross suggests that the primary compositional effects are captured by the manner in which formulations set η_0_ and the extent and position of the shear-thinning regime.

In the context of the analysis, psyllium content emerged as the predominant factor influencing zero-shear viscosity as shown in the following equation:

(9) $$\eta_{0}(Pa\cdot s)=333-210\times {CaCl}_{2}+484\times Psy-210\times {CaCl}_{2}\times Psy+291\times {Psy}^{2}$$

**Figure 8:**
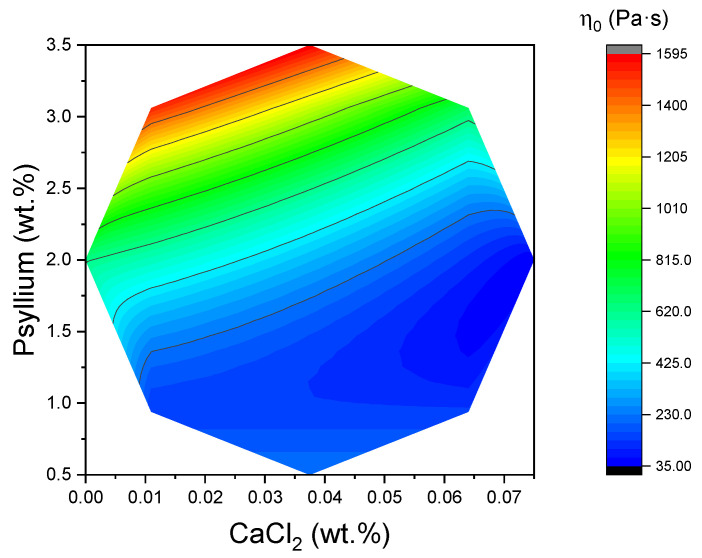
Two-dimensional response surface of zero-shear viscosity (η_0_) values as a function of CaCl_2_ concentration and psyllium.

The incorporation of psyllium resulted in an increase in apparent viscosity across the entire range of shear rates. This observation is indicative of a psyllium-induced densification and cohesion within the network, thereby enhancing its structural integrity. Conversely, the role of CaCl_2_ was secondary. In the context of a constant psyllium level (the coded center for psyllium), it was demonstrated that 0% (*w*:*w*) CaCl_2_ and 2% (*w*:*w*) psyllium exhibited higher viscosity than their counterparts with 0.0375% (*w*:*w*) or 0.075% (*w*:*w*) CaCl_2_, which showed very similar flow curves to each other. Consequently, within the experimental domain, the addition of Ca^2+^ appeared to decrease viscosity in comparison to the Ca-free formulation, irrespective of the psyllium level (see Figure 8). The response surface of the η_0_ mapped over the Central Composite Design (CCD) exhibited a clear maximum in the region of high psyllium and low CaCl_2_, thereby mirroring the qualitative trends seen in the flow curves. The fitted coefficients indicated a strong positive effect of psyllium and a negative effect of Ca^2+^ on η_0_ within the studied ranges, placing the ridge of highest zero-shear viscosity towards the upper-psyllium/lowest-calcium corner of the design space. This topography corresponds with the previously established viscoelastic maps, with the maximum viscosity, η_0_, coinciding with the compositional region exhibiting higher G′ in the LVR and lower tan δ (indicating a stronger gel-like character). This suggests an internal consistency between the small-amplitude (structure-dominated) and steady-shear (flow-dominated) descriptions of the same microstructure.

From a physical-stability standpoint, the combination of a finite critical stress at rest (τ_c_, established from stress sweeps) with a high η_0_ is advantageous for suppressing sedimentation or phase separation during storage. Formulations with higher psyllium (and particularly the Ca-free variants at a given psyllium dose) therefore offer the most favorable baseline for shelf stability. Despite the fact that the four parameters of Cross’s model were mathematically adjusted to a quadratic model using the response surface methodology, only the adjustment of zero Newtonian viscosity (η_0_) to calcium chloride and psyllium concentrations demonstrated an acceptable regression coefficient greater than 0.80 [25], specifically R^2^ = 0.94.

## 3. Conclusions

This work demonstrates that the structure and functionality of kefir can be rationally engineered by integrating fermentation optimization with formulation design. Utilizing a factorial design, the study ascertained the role of inoculum level, temperature, and time in regulating acidification kinetics in whole and skimmed milk. The research identified operating conditions that consistently achieve target pH/TA endpoints, exhibiting discernible matrix-dependent effects between the two milks. Utilizing these conditions as a foundation, a response-surface approach was employed to delineate the individual and interactive roles of psyllium and calcium chloride on small-amplitude viscoelasticity and steady-shear behavior. This approach resulted in the identification of compositional windows that facilitate a balance between gel strength, viscoelastic balance, and processability. Within the explored ranges, psyllium was found to primarily determine network stiffness and zero-shear viscosity, while calcium chloride modulated the gel through ionic interactions, yielding synergistic enhancements only in specific regions of the design space. The resulting low-fat kefir gels exhibited shear-thinning profiles compatible with mixing, pumping, and filling, thereby supporting their technological feasibility. In this study, the definition of “spoonable kefir gels” was directly connected to their rheological properties. The term “spoonability” was identified as being associated with systems in which the elastic component predominated over the viscous one, thereby ensuring firmness while maintaining moderate critical stress and viscosity values that allow for easy deformation under spoon handling. Within the explored formulations, this balance was achieved mainly at higher psyllium and lower CaCl_2_ concentrations, providing practical guidelines for developing kefir gels with the desired spoonable texture.

The proposed strategy is notable for its use of a clean-label approach, the employment of statistically grounded models to guide formulation, and its capacity for direct comparability across milk matrices. The limitations of the present study are as follows: firstly, the bounded factor ranges; secondly, the need to validate long-term stability, microstructure–rheology relationships, and sensory attributes under storage and post-processing conditions. The study as a whole provides actionable guidelines for the development of synbiotic, spoonable kefir gels.

## 4. Materials and Methods

### 4.1. Materials

In all experiments, whole and skimmed cow’s milk (Hacendado brand, Córdoba, Spain) were used as the base for kefir production. As indicated by the manufacturer’s label, the fat content of whole and skimmed milk are 3.6 and 0.3 g/100 mL respectively. The carbohydrate content is 4.9 g/100 mL, of which 4.9 g/100 mL are sugars. The protein content is 3.2 g/100 mL, and the salt content is 0.13 g/100 mL. The calcium content is 120 mg/100 mL. The kefir grains used in this study were obtained from a commercial supplier, Kefiralia (Arrasate, Spain). The granules are composed of a symbiotic consortium of lactic acid bacteria and yeasts embedded in a polysaccharide-protein matrix (primarily kefiran). Prior to experimentation, the grains were acclimatized separately to skimmed milk environment over six fermentation cycles at room temperature (18–20 °C), with milk renewals every 36 h, until stable pH values were achieved in each system. Psyllium husk powder was used as a thickening and prebiotic agent. The commercial product employed was Psyllium Bio El Granero Integral (El Granero, Madrid, Spain), which is derived from the milled outer husk of *Plantago ovata* seeds. The product has been certified as both organic and gluten-free, and is marketed for use in culinary applications. The composition of the substance under scrutiny is primarily soluble fiber, with arabinoxylans being of particular note due to their gelling properties. Calcium chloride dihydrate (CaCl_2_ · 2 H_2_O) was utilized as a structural enhancer and purchased from PanReac AppliChem (ITW Reagents, Barcelona, Spain).

### 4.2. Methods

#### 4.2.1. Kefir Development and Optimization of the Fermentation Process

The initial phase of the study aimed to determine the optimal fermentation conditions for producing kefir from commercial whole and skimmed cow’s milk. Three experimental variables were evaluated: kefir grain inoculum concentration (g) fermentation temperature (T) and fermentation time (t). A factorial design with three variables and three levels (−1, 0, and +1) was employed to assess the individual and interactive effects of these variables on key physicochemical parameters of the final product: pH and total acidity (TA). A total of 10 experimental runs were carried out (see Table 3) All fermentations were conducted in 250 mL glass containers filled with 180 g of commercial skimmed milk, previously brought to ambient temperature. The appropriate amount of kefir grains was added to each container, and samples were then incubated in a thermostatic water bath (JP Selecta Frigiterm, Barcelona, Spain) under static conditions at the target fermentation temperature for the designated time.

Following fermentation, kefir grains were separated from the fermented matrix using a sterile stainless-steel sieve. The pH of the resulting kefir was measured immediately using a calibrated bench pH meter (XS Instruments pH 50 VioLab, Labprocess, Barcelona, Spain). Total acidity was determined by titrating 10 mL of each sample with 0.1 M NaOH until reaching pH 8.4, and results were expressed as g·L^−1^ lactic acid. All measurements were performed in triplicate. The experimental data were fitted to second-order polynomial models to describe the influence of the three variables on pH and TA.

#### 4.2.2. Gel Formation and Rheological Characterization

To evaluate the influence of psyllium and calcium chloride on the rheological properties of skimmed milk kefir, a central composite design (CCD) was applied. This second design was based on fermentation parameters optimized in a previous stage (fermentation temperature of 26 ± 0.3 °C, 24-h duration, and 5.5% (*w*:*w*) kefir grains), which yielded kefir samples with suitable pH and acidity values for subsequent analysis.

The CCD included ten experimental runs, varying two independent variables: psyllium concentration (0.5–3.5% (*w*:*w*) of kefir) and calcium chloride concentration (0.01–0.075% (*w*:*w*) of CaCl_2_). A total of five levels (−1.41, −1, 0, +1, and +1.41) were tested for each factor, including axial and central points, to allow second-order modeling of the responses. The coded and real values of the design are shown in Table 4:

Each formulation was prepared by fermenting 180 g of commercial skimmed milk with 9.9 g of pre-acclimatized kefir grains (5.5% (*w*:*w*)) for 24 h at 26 °C in a thermostatic water bath. After fermentation, the grains were removed, and 150 g of kefir were collected for analysis. Psyllium and CaCl_2_ were added to each sample at the designated concentrations. Psyllium was dispersed using an Ika-Visc MR-D1 homogenizer (IKA, Staufen, Germany) in a 250 mL glass beaker containing 150 g of kefir at 25 ± 1 °C. The sample was stirred at a rate of 400 rpm throughout the addition process. Psyllium was then sprinkled gradually over a period of approximately two minutes in order to promote wetting and avoid lump formation. This process was followed by a further three minutes of stirring. Calcium chloride was pre-dissolved as a 10% (*w*:*w*) aqueous solution and added dropwise over a period of 30 s under constant 400 rpm stirring, followed by an additional 60 s of mixing. It is evident that meticulous consideration was given to the avoidance of a deep vortex, with the objective of minimizing air entrainment. Subsequent to the mixing stage, a period of rest was allocated to all samples, with a duration of 45 min at ambient temperature, with the objective of facilitating the completion of hydration prior to the commencement of rheological testing. To ensure consistent rheological behavior, preliminary tests were conducted to identify the minimum hydration time required after psyllium addition. A frequency sweep was performed at different time intervals on a sample containing 2% (*w*:*w*) psyllium and 0.0375% (*w*:*w*) of CaCl_2_. The test was conducted at a constant shear stress of 0.2 Pa, within the linear viscoelastic region (LVR). No significant changes in viscoelastic parameters (G′ and G″) were observed after 45 min, confirming that this hydration period was sufficient for all subsequent samples.

Rheological measurements were performed using a controlled-stress rotational rheometer (Thermo Scientific™ Haake MARS, Waltham, MA, USA) equipped with parallel plate geometry (35 mm diameter, 1 mm gap), operating at a constant temperature of 25 ± 0.1 °C. Prior to each test, all samples were gently homogenized and allowed to rest to eliminate air bubbles. Three types of tests were conducted to characterize the viscoelastic and flow behavior of kefir samples enriched with psyllium and calcium chloride. First, stress sweep tests were carried out at a fixed angular frequency of 1 Hz to determine the linear viscoelastic region (LVR). The applied shear stress was gradually increased from 0.01 Pa up to values that caused a breakdown in structure. The end of the LVR was defined as the point at which the storage modulus (G′) began to decrease significantly, indicating the critical stress (τ_c_) at which the gel network starts to fail irreversibly. This parameter was used as an indicator of the structural robustness of each formulation.

Subsequently, frequency sweep tests were performed within the LVR, using a constant shear stress of 0.2 Pa. Angular frequency ranged from 0.1 to 10 Hz. For each sample, the elastic modulus (G′), viscous modulus (G″), and loss tangent (tan δ = G″/G′) were recorded. The values of G′ and tan δ provided insight into the gel-like behavior of the samples, with tan δ < 0.1 indicative of strong gels, intermediate values suggesting weak gels or viscoelastic pastes, and values greater than 1 denoting a predominantly viscous behavior. Additionally, flow behavior was assessed through steady shear tests in which shear rate was increased in steps up to 30 s^−1^. Flow curves (viscosity vs. shear rate) were fitted to the Cross model to describe the non-Newtonian, shear-thinning behavior observed. The Cross model parameters, that are zero-shear viscosity (η_0_), consistency index, and flow index, were calculated to quantify and compare the flow profiles between samples.

All rheological measurements were carried out in duplicate for each formulation. Moreover, the central point of the was replicated three times to evaluate experimental variability and ensure model robustness. The rheological parameters obtained (G′ within the LVR, critical stress τc, tan δ, and flow curve fit data) were analyzed using response surface methodology (RSM) to assess the individual and interactive effects of both variables. Statistical modeling was conducted using Echip software 7 (Experimentation by Design, Wilmington, DE, USA), and the quality of fit and significance of effects were evaluated at the 95% confidence level. Surface plots and predictive models were generated to identify optimal conditions for gel strength, viscoelasticity, and flow resistance.

#### 4.2.3. Statistical Analysis

All experiments were performed at least in duplicate, and data are reported as the mean. One-way ANOVA was used to evaluate significant differences among treatments, with *p* < 0.05 considered statistically significant. The fitting of rheological and kinetic data was carried out using Origin software 2021 (OriginLab Corporation, Northampton, MA, USA).

## Figures and Tables

**Table 1:** Total acidity (TA, expressed as g lactic acid·L^−1^) and pH values for all experiments performed as a function of fermentation time and temperature and kefir nodule concentration.

Experiment	Skimmed Milk		Whole Milk	
	pH	TA (g/L)	pH	TA (g/L)
1	5.94	1.56	6.12	1.5
2	5.60	2.64	5.67	2.73
3	5.26	4.05	5.47	2.73
4	4.60	5.92	4.84	5.92
5	5.04	5.99	4.78	5.40
6	3.93	12.11	3.99	9.55
7	3.98	10.03	3.87	11.65
8	3.56	19.34	3.54	22.1
9	4.27	6.85	4.28	7.81
10	4.27	6.46	4.23	8.32

**Table 2:** Values of critical stress (τ_c_), elastic modulus at the linear viscoelastic range (G′_LVR_), tan δ at 1 Hz and zero-shear viscosity (η_0_) for the experimental design used in the rheological characterization.

Experiment	CaCl_2_ Concentration (% (*w*:*w*))	Psyllum Concentration (% (*w*:*w*))	τ_c_ (Pa)	G′_LVR_ (Pa)	tan δ	η_0_ (Pa·s)
1	0.0375	2.00	1.07	26.7	0.35	104
2	0.0375	2.00	1.74	95.6	0.29	655
3	0.0000	2.00	6.95	294.7	0.27	1740
4	0.0750	2.00	5.85	132.3	0.27	607
5	0.0375	0.50	2.89	113.4	0.28	347
6	0.0375	3.50	3.01	113.8	0.28	491
7	0.0110	0.94	0.64	17.3	0.33	163
8	0.0110	3.06	10.42	209.6	0.25	1589
9	0.0640	0.94	3.68	132.6	0.26	342
10	0.0640	3.06	3.39	125.6	0.27	312

**Table 3:** Values of independent variables for the experimental design used in the optimization of the fermentation process.

Experiment	Kefir Grain Inoculum Concentration (% (*w*:*w*))	Fermentation Temperature (°C)	Fermentation Time (h)
1	1	20	12
2	10	20	12
3	1	26	12
4	10	26	12
5	1	20	48
6	10	20	48
7	1	26	48
8	10	26	48
9	5.5	23	30
10	5.5	23	30

**Table 4:** Values of independent variables for the experimental design used in the gelification and rheological characterization.

Experiment	CaCl_2_ Concentration (% (*w*:*w*))	Psyllum Concentration (% (*w*:*w*))
1	0.0375	2.00
2	0.0375	2.00
3	0.0000	2.00
4	0.0750	2.00
5	0.0375	0.50
6	0.0375	3.50
7	0.0110	0.94
8	0.0110	3.06
9	0.0640	0.94
10	0.0640	3.06

## Data Availability

The raw data supporting the conclusions of this article will be made available by the authors on request.

## Abbreviations

The following abbreviations are used in this manuscript:

TA	Total acidity
LVR	Linear viscoelastic range
RSM	Response Surface Methodology
CCD	Central Composite Design
